# Non-tuberculous *mycobacteria* enhance the tryptophan-kynurenine pathway to induce immunosuppression and facilitate pulmonary colonization

**DOI:** 10.3389/fcimb.2024.1455605

**Published:** 2024-10-21

**Authors:** Longjie Li, Jiaofang Shao, Chunran Tong, Weiwei Gao, Pan Pan, Chen Qi, Chenxi Gao, Yunlei Zhang, Ying Zhu, Cheng Chen

**Affiliations:** ^1^ Department of Respiratory and Critical Care Medicine, The Affiliated Jiangning Hospital of Nanjing Medical University, Nanjing Medical University, Nanjing, China; ^2^ School of Biomedical Engineering and Informatics, Nanjing Medical University, Nanjing, China; ^3^ Department of Tuberculosis, The Second Hospital of Nanjing, Nanjing University of Chinese Medicine, Nanjing, China; ^4^ Department of Cardiothoracic Surgery, Jinling Hospital, Affiliated Hospital of Medical School, Nanjing University, Nanjing, China; ^5^ Department of Infectious Disease, The Affiliated Jiangning Hospital of Nanjing Medical University, Nanjing Medical University, Nanjing, China

**Keywords:** non-tuberculous mycobacterium, tuberculosis, lung microbiota, microbial interaction, bronchoalveolar lavage fluid, immunosuppressive milieu

## Abstract

The increasing prevalence of non-tuberculous mycobacterium (NTM) infections alongside tuberculosis (TB) underscores a pressing public health challenge. Yet, the mechanisms governing their infection within the lung remain poorly understood. Here, we integrate metagenomic sequencing, metabolomic sequencing, machine learning classifiers, SparCC, and MetOrigin methods to profile bronchoalveolar lavage fluid (BALF) samples from NTM/TB patients. Our aim is to unravel the intricate interplay between lung microbial communities and NTM/*Mycobacterium tuberculosis* infections. Our investigation reveals a discernible reduction in the compositional diversity of the lung microbiota and a diminished degree of mutual interaction concomitant with NTM/TB infections. Notably, NTM patients exhibit a distinct microbial community characterized by marked specialization and notable enrichment of *Pseudomonas aeruginosa* and *Staphylococcus aureus*, driving pronounced niche specialization for NTM infection. Simultaneously, these microbial shifts significantly disrupt tryptophan metabolism in NTM infection, leading to an elevation of kynurenine. *Mycobacterium intracellulare*, *Mycobacterium paraintracellulare*, *Mycobacterium abscessus*, and *Pseudomonas aeruginosa* have been implicated in the metabolic pathways associated with the conversion of indole to tryptophan via tryptophan synthase within NTM patients. Additionally, indoleamine-2,3-dioxygenase converts tryptophan into kynurenine, fostering an immunosuppressive milieu during NTM infection. This strategic modulation supports microbial persistence, enabling evasion from immune surveillance and perpetuating a protracted state of NTM infection. The elucidation of these nuanced microbial and metabolic dynamics provides a profound understanding of the intricate processes underlying NTM and TB infections, offering potential avenues for therapeutic intervention and management.

## Introduction

Non-tuberculous mycobacteria (NTM) are highly prevalent in soil and water ecosystems, and their incidence in humans has risen due to factors such as the ageing of the population, the prevalence of lung diseases, the overuse of antibiotics, and the improvement in microbiologic diagnostic techniques (Naito et al., 2020; Wang et al., 2023). Notably, the global burden of NTM infections in developed countries now surpasses that of new tuberculosis (TB) infections (Furuuchi et al., 2019; Winthrop et al., 2020). In contrast to TB, NTM diseases pose greater therapeutic challenges and are associated with a higher frequency of toxicity-related complications. Indeed, 90% of immunocompetent individuals exposed to NTM/MTB do not develop active disease (Sulaiman et al., 2018; Varley and Winthrop, 2022). The infection individuals are thought to be acquired via a host response—the interaction between an individual’s distinct lung microbiome and NTM (Gupta et al., 2018). Therefore, an in-depth exploration of the interplay between NTM/TB and lung microbiota holds the potential to advance our comprehension of NTM and TB infections, thus offering novel insights into strategies for disease prevention and treatment.

Multiple studies have demonstrated the importance of the interaction between the microbiome, its metabolites, and the host innate immune system in maintaining organismal homeostasis (Gupta et al., 2018; Varley and Winthrop, 2022). The current understanding of changes in the lung microbiome induced by TB/NTM infections primarily relies on the studies involving sputum and bronchoalveolar lavage fluid (BALF) (Krishna et al., 2016; Wang and Xing, 2023; Zhou et al., 2015). For example, bacterial diversity was significantly higher among sputum isolates of TB patients than of healthy controls while healthy participants demonstrated a strong clustering pattern in comparison with the TB patients had a more scattered pattern (Cui et al., 2012). Additionally, sputum analysis suggested that the presence of foreign bacteria and changes in lung microbiome are not only associated with the onset of TB infection but also with the recurrence and failure of therapy (Wu et al., 2013). *Mycobacterium* tuberculosis (MTB) dominates microbiota in lung samples from TB patients, thus changing the microbial community structure for their colonization (Hu et al., 2020). NTM shares common bacterial components and antigens with Mycobacterium tuberculosis, but demonstrates relatively subdued virulence. The sputum and BALF microbiota from NTM patients tended to have fewer operation taxonomy units (OTUs), Shannon evenness, and β-diversity compared with those from the control group (Choi et al., 2023; Kang et al., 2021). In addition, BALF samples from NTM-positive patients were occupied with *Oxalobacteraceae*, while the samples from NTM-negative ones were enriched with *Porphyromonas* (Sulaiman et al., 2018). Also, the NTM-positive patients contain a significantly high rate of anaerobes compared with the bronchiectasis patients without NTM (Yamasaki et al., 2015).

In addition, microbial metabolic activity and their products may also influence the NTM/MTB infection. For example, there was more production of short-chain fatty acids such as butyric and propionic acids in the HIV infected individuals with active TB (Lawani and Morris, 2016). MTB infection could increase the production of Butyrate to restrain mycobacterial antigen-specific IL-17 and IFN-γ responses, thus increasing MTB antigen-specific FOXP3+ regulatory T cells in the lungs (Lachmandas et al., 2016; Segal et al., 2017). Also, several metabolites significantly differed in the NTM patients compared to controls, including decreased levels of tryptophan-associated and branched-chain amino acid metabolites, including itaconate, ceramides (C_18_ backbones), 2-methylcitrate/homocitrate, and intermediates in the utilization of both aromatic and branched-chain amino acid metabolism, and the increased levels of compounds involved in phospholipid metabolism (Breen et al., 2022). These findings demonstrated that MTB and NTM could regulate the lung microbiota and alter functional gene expression to regulate immune response, thus establishing beneficial niche for their survival. However, until now, most findings come from the studies of TB patients and sputum samples. The precise molecular mechanisms underlying the host’s immune response against NTM/TB remain incompletely understood.

Recent advances in high-throughput sequencing, including metagenomics and metabolomics, offer a promising avenue for analyzing microbial communities and metabolic profiles in respiratory infections. Multi-Omic analysis can provide us with more insights into how MTB regulates the lung microbiota and interacts with the immune system. Specially, no metagenomic analysis has been conducted on the BALF samples of NTM patients. Therefore, we aim to combine metagenomic and metabolomic analyses to uncover unique microbial and metabolic characteristics of NTM and TB infections, thereby understanding the interactions of lung microbiota and their metabolites in TB and NTM infections, thus improving diagnostic accuracy, optimizing treatments, and enhancing patient outcomes in managing NTM and TB infections.

## Materials and methods

### Subject recruitment

The subjects were recruited based on the presence of respiratory system symptoms and radiological abnormalities consistent with NTM and TB infection from The Affiliated Jiangning Hospital of Nanjing Medical University. Approval for the study was obtained from the Ethics Committee of The Affiliated Jiangning Hospital of Nanjing Medical University (Approval No: IACUC-2212016), and informed consent was obtained from all participants. Clinical diagnoses were established through various methods, including acid-fast bacilli smear, bronchoscopy, bronchoalveolar lavage fluid (BALF) mycobacterial culture, and comprehensive radiological analysis. TB-positive patients were defined as those who tested positive for acid-fast bacilli on sputum smear examination, exhibited clinical manifestations resembling pulmonary tuberculosis, and had at least one positive result for MTB in the BALF. NTM-positive patients were defined as those who tested positive for acid-fast bacilli on sputum smear examination, exhibited clinical manifestations distinct from pulmonary tuberculosis, and had at least one positive result for one of the 168 non-tuberculous mycobacterial species in the BALF. In total, BALF samples were collected from 121 subjects, comprising 96 samples for metagenomic analysis and 25 samples for untargeted metabolomic analysis. These samples were obtained from a cohort of 96 participants, including 51 TB patients, 38 NTM patients, and 7 healthy controls.

### Sample isolation and library preparation

Nucleic acid analysis and sequencing in this investigation were conducted utilizing Illumina’s HISEQ4000 platform. A volume of 5 mL BALF was collected and processed using the HostZerotm Microbial DNA Kit (D4310, Zymo Research). Commercial library preparation reagents intended for Illumina sequencing systems were employed to generate the libraries. The libraries were assembled using the Kapa HyperPlus Library Preparation Kit (kk8514, Kapa). Fragment lengths within the libraries were assessed using the Agilent 2100 Bioanalyzer, while library concentrations were determined using the Qubit dsDNA HS Assay Kit (Q32854, Thermo Fisher Scientific). Ultimately, sequencing analysis was performed on the HISEQ4000 platform (Illumina).

### Sequencing data processing and analysis

We utilized the Fastp software (v0.23.4) to scrutinize the raw sequencing data, thereby obtaining a set of superior-quality sequencing data. To validate the accuracy of the microbial data, we assessed host background levels through alignment of the filtered reads to the human genome using the bowtie2 (v2.5.1). Reads that did not map to the host genome were subjected to taxonomic classification via the Kraken2 (v2.1.3). The quantification of taxonomy abundances at the species level was executed utilizing Bracken. Furthermore, to decipher the metabolic contributions embedded within the samples, we harnessed the capabilities of the HMP Unified Metabolic Analysis Network (HUMAnN3). The fractions of gene families were unveiled through the utilization of HUMAnN3. These quantifications were subsequently aligned with microbial pathways and elevated-level categories, guided by the pathway hierarchy derived from the MetaCyc/KEGG metabolic pathway database.

### Analysis of microbial co-abundance networks

To delve into the intricate network of species associations, the SparCC algorithm was employed to compute correlations among microbial entities. The statistical significance of notable co-abundance patterns was rigorously maintained at a threshold of R > 0.4 and *p* < 0.05, a control achieved through the execution of 100 permutations. Subsequent to these analytical steps, the R package ggcluster was adeptly utilized to both arrange the network components and produce the visual representation.

### Untargeted metabolomics analysis of BALF samples

A total of 25 BALF samples (including 6 healthy controls, 5 NTM patients, and 14 TB patients) were subjected to untargeted metabolomics analysis. The samples were thawed at 4°C, vortex-mixed, and 100 μL of each sample was transferred to EP tubes. To each sample, 400 μL of pre-cooled methanol solution was added, mixed, followed by a 20 min ultrasonication in an ice bath. After this, the samples were allowed to stand at -20°C for 1 h, followed by centrifugation at 16,000 ×g and 4°C for 20 min. The supernatant was collected, vacuum-concentrated, and reconstituted with 100 μL of methanol-water solution (1: 1, v/v). The reconstituted samples were centrifuged at 20,000 ×g and 4°C for 15 min, and the supernatants were subjected to mass spectrometry analysis.

Throughout the analysis, samples were maintained at 4°C within an automated autosampler. An SHIMADZU-LC30 ultra-high-performance liquid chromatography system (UHPLC) was employed, equipped with an ACQUITY UPLC^®^ HSS T3 column (2.1 × 100 mm, 1.8 µm) from Waters, Milford, MA, USA. The injection volume was 4 μL, column temperature was set to 40°C, BALF and flow rate was maintained at 0.3 mL/min. The chromatographic mobile phases consisted of A: 0.1% formic acid aqueous solution and B: acetonitrile. The gradient elution program was as follows: 0 - 2 min, 0% B; 2 - 6 min, linear increase of B from 0% to 48%; 6 -10 min, linear increase of B from 48% to 100%; 10-12 min, B held at 100%; 12 - 12.1 min, linear decrease of B from 100% to 0%; 12.1 - 15 min, B held at 0%.

Each sample underwent electrospray ionization (ESI) in both positive and negative ion modes. Post-UPLC separation, a QE Plus mass spectrometer (Thermo Scientific) was employed for mass spectrometry analysis. HESI source ionization conditions included Spray Voltage: 3.8 kV (+) and 3.2 kV (-); Capillary Temperature: 320°C (±); Sheath Gas: 30 (±); Aux Gas: 5 (±); Probe Heater Temp: 350°C (±); S-Lens RF.

Raw data underwent peak alignment, retention time correction, and peak area extraction using the MSDIAL software. Metabolite identification employed both accurate mass matching (with mass tolerance < 10 ppm) and secondary spectrum matching (with mass tolerance < 0.01 Da). Public databases such as HMDB, MassBank, and GNPS were queried for identification. Data integrity was upheld by discarding ion peaks with > 50% missing values within each group. Positive and negative ion data were normalized based on total peak area, integrated, and subjected to pattern recognition through Python software. Data were preprocessed with Unit Variance scaling (UV) prior to subsequent analysis.

### Random forest classifier

We developed a classifier using a random forest model (randomForest 4.6 - 14 package) to distinguish samples from healthy controls, TB, and NTM patients based on the relative abundances of differential microbes. To evaluate the performance of the predictive model, we employed a 10-fold cross-validation approach, conducting five trials. To prevent overfitting, we performed feature selection on the model’s hyperparameters. Specifically, all microbes were ranked by their variable importance, and the top 50 species were incrementally added to the model. As the number of species increased, the mean cross-validation error was calculated. The cutoff for feature selection was defined as the minimum error on the average curve plus the standard deviation at that point. All sets with errors below this were listed, and the set with the fewest species was chosen as the optimal set. Receiver operating characteristic (ROC) curves were plotted for both the training and validation cohorts, and the area under the curve (AUC) was calculated using the pROC package.

### Statistical analysis

All statistical analyses were conducted using R (v4.3.1). Differential relative abundances of taxa were assessed using the LEfSe algorithm, which employs the Kruskal-Wallis test to detect significant differences, the Wilcoxon test for pairwise comparisons, and Linear Discriminant Analysis (LDA) to estimate effect sizes of differentially abundant OTUs. Differences in α diversity were analyzed using the Kruskal-Wallis test followed by Dunn’s *post-hoc* test, considered significant at FDR-adjusted p < 0.05. The Mann-Whitney U test was applied to evaluate the differential relative abundance of KOs, KO-enriched pathways, metabolites, and metabolite-enriched pathways, considered significant at FDR-adjusted p < 0.05. Enrichment in healthy controls, TB, and NTM patients was identified based on higher mean U values. At the microbial species level, PERMANOVA based on Bray-Curtis distances, with 9,999 permutations, was used to assess the impact of microbial composition on disease states, and the resulting dissimilarity profiles were visualized using Principal Coordinates Analysis (PCoA). Metabolomic composition differences related to disease states were evaluated through metabolite PLS-DA, with p-values obtained via permutation testing. Spearman rank correlation coefficients were calculated to explore associations between the relative abundances of differential species and metabolites across healthy controls, TB, and NTM patients, considered significant at FDR-adjusted p < 0.05. All p-values were adjusted using the Benjamini-Hochberg method to control the false discovery rate (FDR).

## Results

### Characterization of BALF microbiome

**Figure 1:**
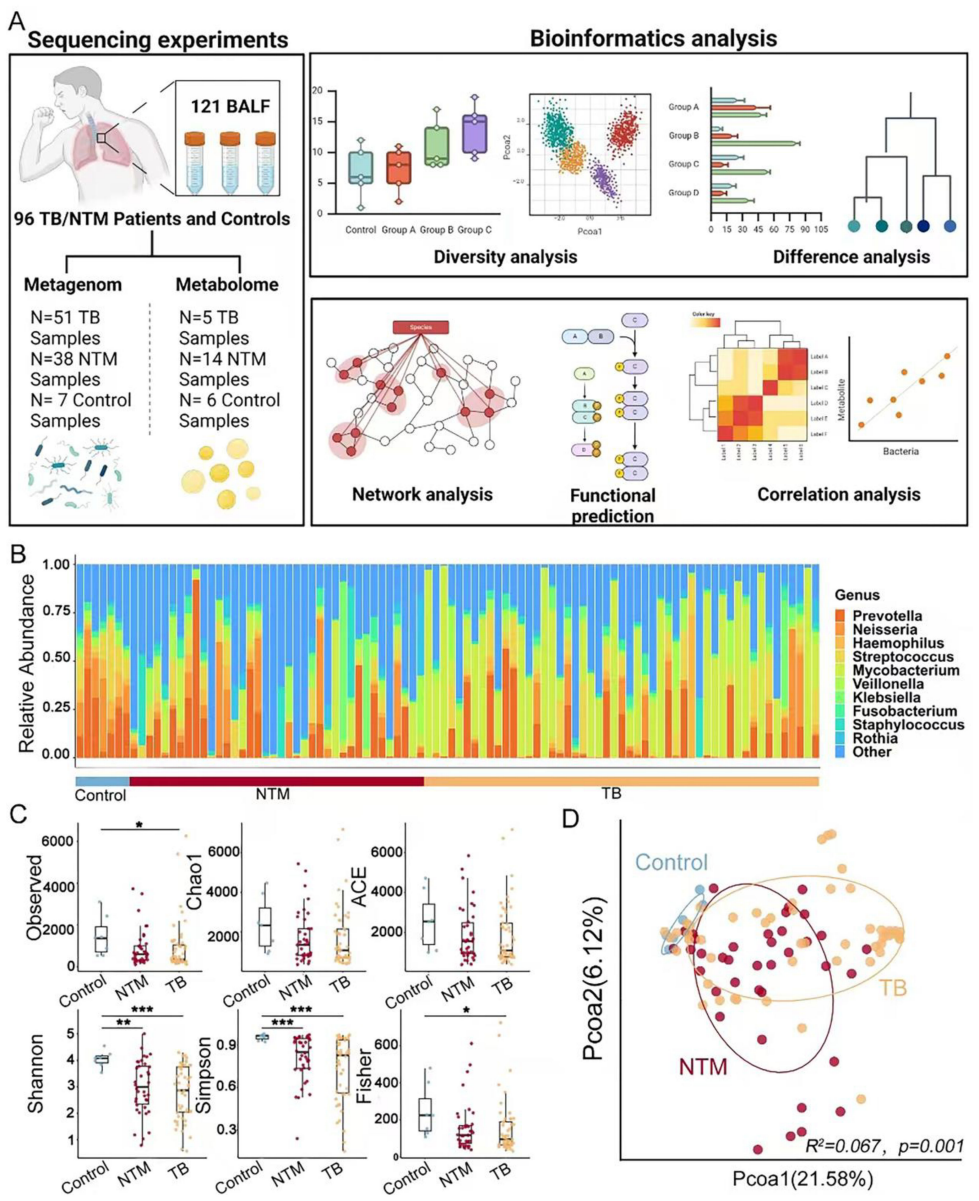
Characterization of BALF microbiome. **(A)** Workflow for cross-cohort integration analysis of fecal metagenomics and metabolomics in BALF. **(B)** Genus-level distribution of bacteria in the BALF microbiota of TB/NTM patients (n = 89) and healthy controls (n = 7). **(C)** Distribution of microbiota α diversity (Observed, Shannon, Fisher, Simpson, Chao1 and ACE index) comparing TB/NTM and healthy controls. Significance was confirmed using the Kruskal-Wallis test, followed by Dunn’s *post-hoc* test with FDR-adjusted p-values adjusted using the Benjamini-Hochberg method for multiple comparisons. **(D)** PCoA plots of microbiota β-diversity (Bray-Curtis dissimilarity metric) comparing TB/NTM and healthy controls. R² and FDR-adjusted p-values, using the Benjamini-Hochberg method, were tested by PERMANOVA. * FDR-adjusted p < 0.05, ** FDR-adjusted p < 0.01, *** FDR-adjusted p < 0.001.

We performed shotgun metagenomic sequencing to analyze the lung microbiota in a total of 96 BALF samples (**Supplementary Table S1**) (**Figure 1A**). In the entire dataset, the phyla *Pseudomonadota, Bacteroidota, Bacillota, Actinomycetota*, and *Fusobacteriota* were found to be the most abundant, comprising 97.93% of the total reads, in both NTM/TB cases and healthy controls. Among the identified 1816 genera, *Prevotella, Neisseria, Haemophilus, Streptococcus*, and *Mycobacterium* were the most abundant, representing 80.4% of the total reads (**Figure 1B**).

Then, we examined whether infections caused by NTM or MTB would affect the microbial community in the lungs. The results showed that the TB group had significantly lower observed index (FDR-adjusted *p* = 0.016), Shannon index (FDR-adjusted *p* = 0.008), Simpson index (FDR-adjusted *p* = 0.0009), and Fisher index (FDR-adjusted *p* = 0.018) compared to healthy controls. Similarly, the NTM group showed significantly lower Shannon index (FDR-adjusted *p* = 0.0029) and Simpson index (FDR-adjusted *p* = 0.0006) compared to healthy controls (**Figure 1C**). These findings indicate that both TB and NTM patients had significantly lower α-diversity compared to healthy controls, suggesting a reduction in the compositional diversity and homogeneous of the lung microbiota due to infection. Importantly, there was no significant difference in α-diversity between NTM and TB patients, indicating similar effects of these infections on the lung microbiota.

To further investigate the clustering patterns of the three microbial communities, PCoA analysis was conducted. Samples clustered within their respective groups and exhibited separation between different groups (**Figure 1D**, R^2^ = 0.067, FDR-adjusted *p* = 0.001). The distribution of NTM and TB samples exhibited relatively greater dispersion when compared to healthy control individuals. The distinct separation of microbial community centers among NTM, TB, and healthy control groups signifies substantial differences in their compositions. However, the shaping of lung microbiota by NTM and TB infections exhibits similarity.

### Differential abundance of bacterial species in the BALF of TB/NTM patients and healthy controls

**Figure 2:**
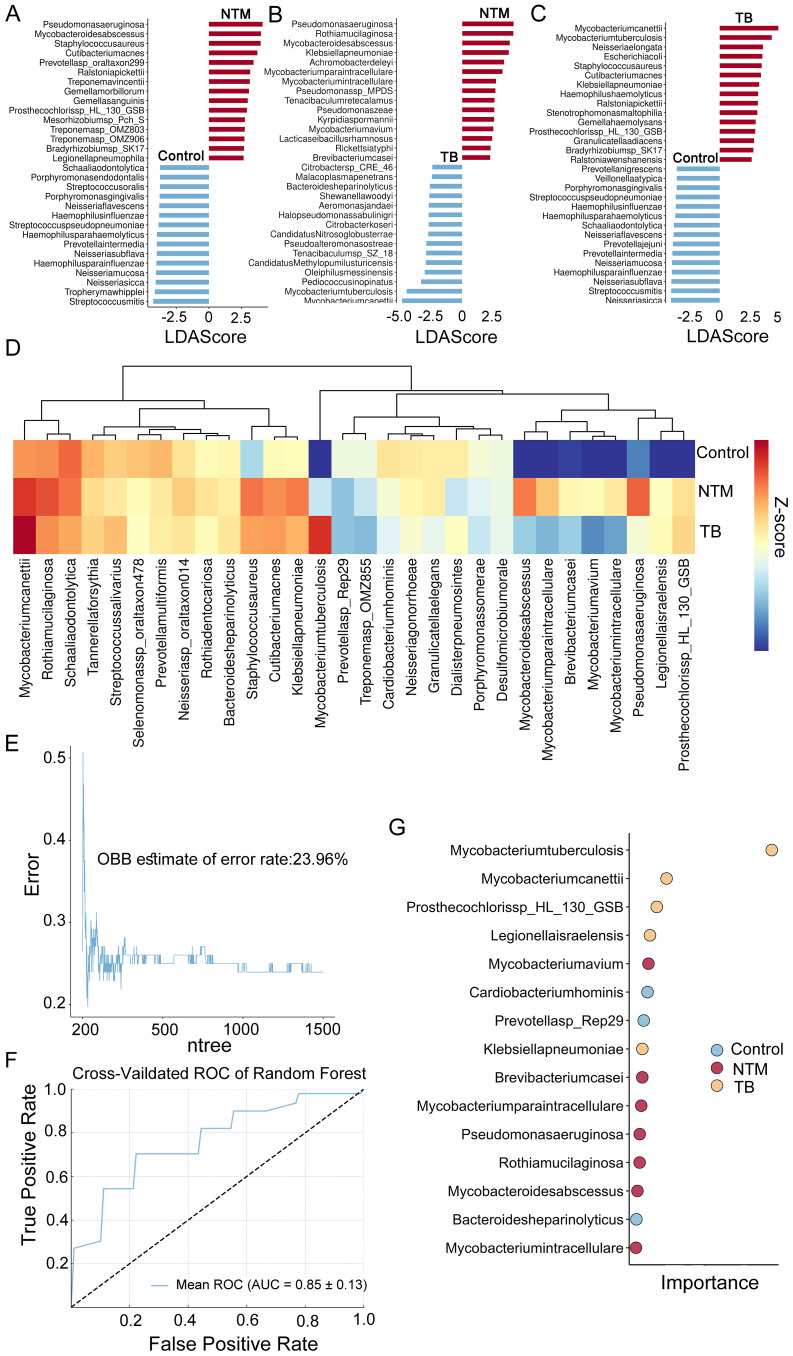
Microbial taxa alterations between TB/NTM and healthy control. **(A-C)** LEfSe analysis identified the microbes whose abundances significantly differed between the NTM and Controls groups **(A)**, the TB and Controls groups **(B)**, as well as between the NTM and TB groups **(C)**. The findings regarding the top 30 species exhibiting abundance differences are depicted in the plot. **(D)** 30 species specifically identified by RF in TB, NTM, and control groups. Heatmaps depict the average relative abundances. **(E)** RF model OBB estimate of the error rate curves for classification of TB, NTM and control based on differentially selected bacterial species (as determined by LEfSe). The model was configured with 1500 trees (ntree) and 30 selected features (nfeatures). **(F)** The RF model yielded a mean AUC of 0.85 with a standard deviation of 0.13 for the ROC curve, obtained through k-fold cross-validation. **(G)** The RF model assessed the importance of 30 specifically selected species, with the dotplot displaying the top 15 species in terms of their importance.

To identify species with differential relative abundance between the NTM, TB, and healthy control microbial communities, we employed the microbiome Linear discriminant analysis Effect Size (LEfSe). A total of 387 microbial species were found to be significant differences (*p* < 0.05, LDA > 2) (**Supplementary Table S2**). Compared to healthy controls, the NTM group showed significant enrichment of 37 species and significant reduction of 189 species. Consistent with previous reports (Johansen et al., 2020), *Mycobacterium abscessus* (*M. abscessus*), *Mycobacterium avium* (*M. avium*), *Mycobacterium paraintracellulare* (*M. paraintracellulare*), and *Mycobacterium intracellulare* (*M. intracellulare*) were significantly enriched. Additionally, we observed substantial enrichment of *Pseudomonas aeruginosa* (*P. aeruginosa*) and *Staphylococcus aureus* (*S. aureus*) in the NTM group (**Figure 2A**). Studies have indicated that *P. aeruginosa* may predispose individuals to *M. abscessus* infection (Johansen et al., 2020). In comparison to healthy controls, the TB group showed significant enrichment of 49 species and significant reduction of 222 species, with 20 species overlapping with the NTM group, suggesting similar trends between NTM and TB. *Mycobacterium canettii* and MTB were the most enriched species in the TB group (**Figure 2B**). The downregulation of many species in both the NTM and TB groups compared to healthy controls is consistent with mentioned above on α diversity. The differential test between NTM and TB groups was also performed to find out the distinct infection characteristics. In addition to the significant enrichment of NTM species, such as *M. abscessus*, *M. avium*, *M. paraintracellulare*, and *M. intracellulare*, there was a significant increase in *P. aeruginosa*, *Rothia mucilaginosa* (*R. mucilaginosa*), and *Klebsiella pneumoniae* (*K. pneumoniae*) (**Figure 2C**), suggesting differences and potential biological significance between the NTM and TB groups.

The significant microbial differences observed among NTM patients, TB patients, and healthy controls prompted us to investigate whether the BALF microbiome could effectively distinguish these groups and identify disease-specific species. Using the expression levels of 387 differentially abundant microbes, we constructed a disease classifier employing a random forest (RF) model (**Supplementary Table S3**) (see methods). The top 50 microbes ranked by feature importance were further included in the disease classifier. After feature selection based on 10-fold cross-validation, a final combination of 30 species was retained for optimal performance (**Figure 2D**), and a classification model was built on this basis. Notably, the out-of-bag error rate estimate was only 23.96% (**Figure 2E**), and the AUC was 0.853 ± 0.13 (**Figure 2F**), indicating a relatively high discriminatory ability. Several species, including *M. avium*, *Brevibacterium casei* (B. casei), *M. paraintracellulare*, *P. aeruginosa*, *R. mucilaginosa*, *M. abscessus*, and *M. intracellulare*, were identified as NTM-specific species, suggesting they may play important roles in disease development (**Figure 2G**).

### Specific interacting microbial communities were enriched in the BALF of TB/NTM and healthy controls

**Figure 3:**
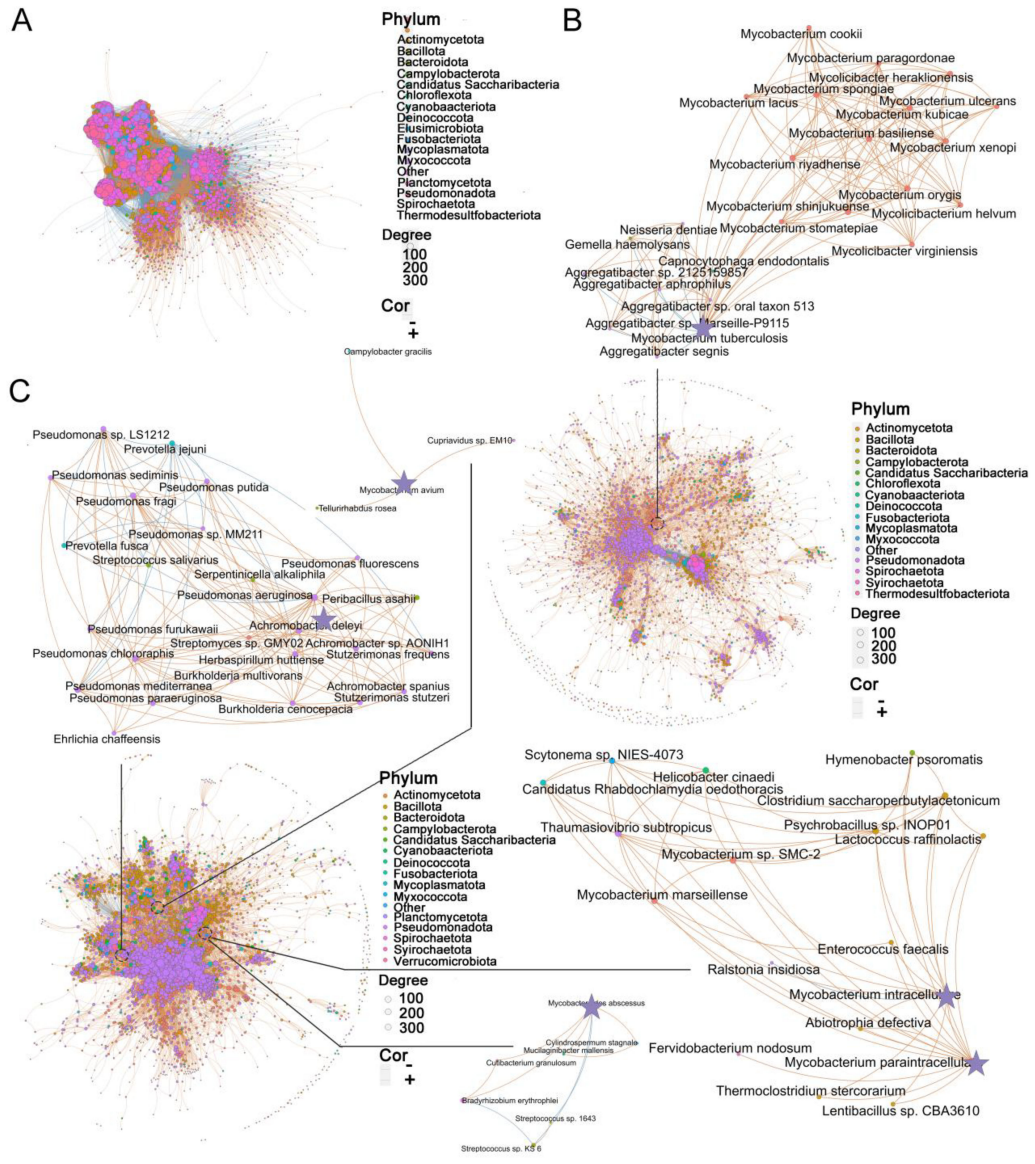
Disease status specific co-abundance species. **(A-C)** Microbial co-abundance communities specific to **(A)** healthy control, **(B)** TB, and **(C)** NTM were identified using SparCC. Arrows point to specific pathogenic species and their associated species within each group. Each node represents an individual species, with node size indicating betweenness centrality and color denoting phylum classification. The connections between nodes illustrate significant species-species co-abundance relationships, with red lines representing positive correlations and blue lines indicating negative correlations. Thicker edges highlight unique connections within each group. SPARCC uses a permutation-based approach to assess the FDR-adjusted *p*.

While diversity metrics and differential abundance analysis can capture broad variations in microbial composition, the lung microbiome represents an ecological community characterized by intricate interactions between resident species. To explore the species-specific differences in interrelationships, we employed the SparCC method. A total of 8,374 species were analyzed, and correlations with R < 0.4 were filtered out (**Supplementary Table S4**). Subsequently, in healthy controls, 4,811 species generated 249,238 significant correlations (**Figure 3A**), whereas in TB patients, 3,088 species generated 43,597 significant correlations (**Figure 3B**), and in NTM patients, 3,746 species generated 65,333 significant correlations (**Figure 3C**) (R *≥* 0.4, FDR-adjusted *p <* 0.05). Compared to the healthy controls, the microbes in the NTM and TB groups exhibited a significantly reduced degree of mutual interaction. Community clusters or modules were identified in both NTM, TB, and healthy controls. The aggregation of species within the same phylum indicated their coordinated response to the environment, and the NTM community exhibited greater specialization.

Subsequently, we focused on examining the microbial interactions involved with the aforementioned 30 significantly different species in each group. In the TB group, 22 species showed correlations with MTB, with 15 species exhibiting positive correlations and 7 species showing negative correlations (**Figure 3B**). Most of the positively correlated species belonged to the *Mycobacterium* genus, including *Mycobacterium basiliense* (*M. basiliense*)*, Mycobacterium riyadhense* (*M. riyadhense*)*, Mycobacterium orygis* (*M. orygis*), among others. Interestingly, the *Aggregatibacter* genus displayed a negative correlation with MTB, suggesting a potential competitive relationship between them. In the NTM group, *M. intracellulare* and *M. paraintracellulare* were frequently co-occurring. *M. intracellulare* had 13 associated species, with 12 displaying positive correlations and 1 showing a negative correlation (**Figure 3C**). Notably, *Mycobacterium marseillense* (*M. marseillense*) exhibited a high correlation with *M. intracellulare.* On the other hand, *M. paraintracellulare* had 16 positively correlated species, most of which were identical to those associated with *M. intracellulare*, except for *Fervidobacterium nodosum* (*F. nodosum*)*, Scytonema* sp. *NIES-4073* (*S.* sp. *NIES-4073*)*, Helicobacter cinaedi* (*H. cinaedi*), and *Rhabdochlamydia oedothoracis* (*R. oedothoracis*), which were exclusive to *M. paraintracellulare*. Fewer species were correlated with *M. abscessus* and *M. avium*, with 5 and 3 species, respectively. *Bradyrhizobium erythrophlei* (*B. erythrophlei*) and *Cutibacterium granulosum* (*C. granulosum*) exhibited higher correlations with *M. abscessus*, while *M. avium* showed weak correlations only with *Tellurirhabdus rosea* (*T. rosea*)*, Cupriavidus* sp. *EM10* (*C.* sp. *EM10*), and *Campylobacter gracilis* (*C. gracilis*). Apart from the NTM bacteria, 25 species were associated with *P. aeruginosa*, with the majority belonging to the *Pseudomonas* genus. *Streptomyces* sp. *GMY02* (*S.* sp. *GMY02*), *Peribacillus asahii* (*P. asahii*), and *Streptococcus salivarius* (*S. salivarius*) were among the species with the highest correlation coefficients. In conclusion, our findings suggest that microbial co-abundance network analysis can identify highly interactive communities that may play a role in influencing health or disease status.

### Divergent bacterial functional metagenomic pathways in BALF of TB, NTM and healthy controls

**Figure 4:**
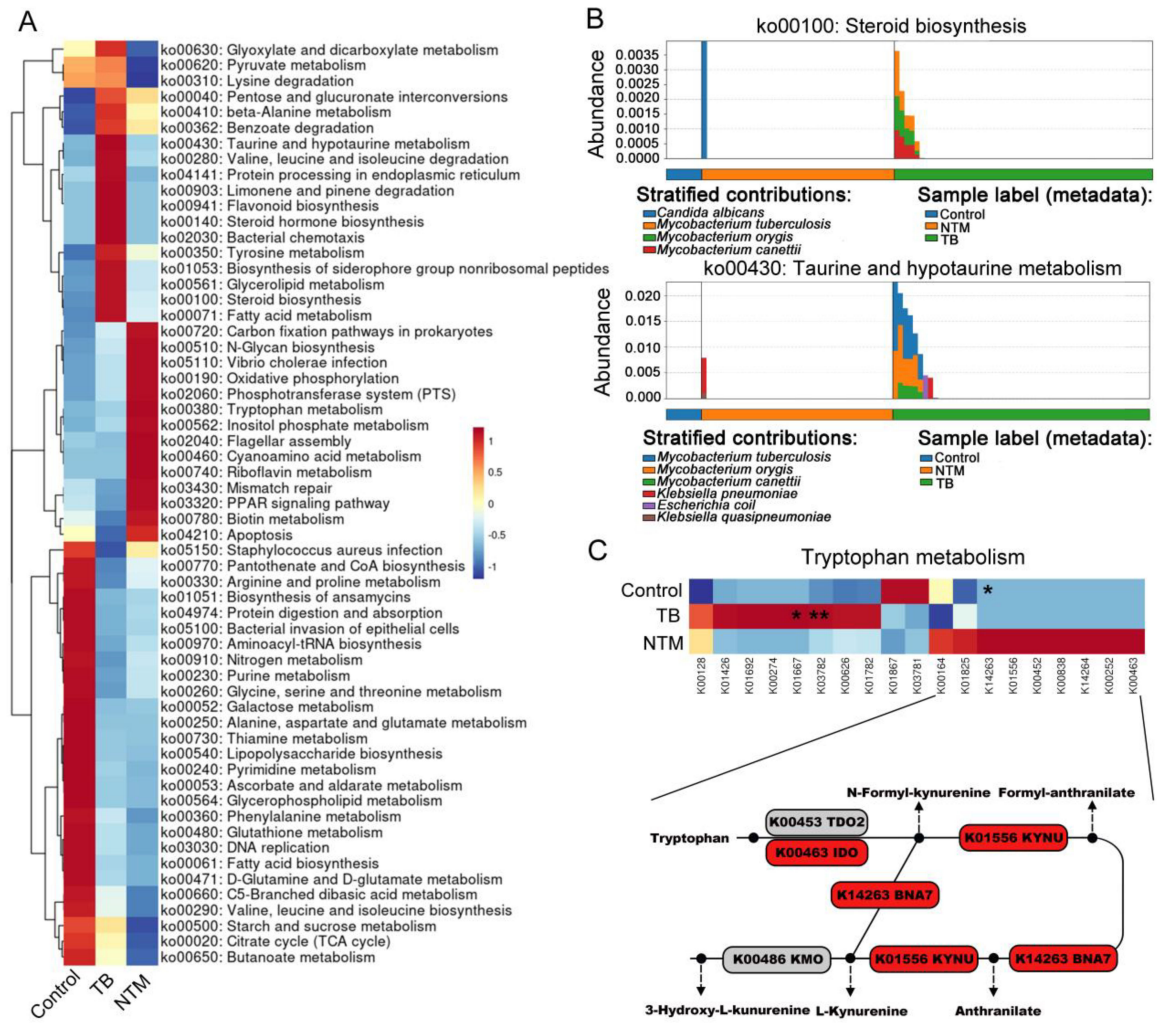
TB/NTM-associated changes in microbial genes summarized in KO genes and KEGG pathway modules. **(A)** The mean abundance of KEGG pathways, differentially enriched in the lung microbiomes of TB, NTM, and control subjects, is presented. The heatmap illustrates the representative microbial KEGG pathways. Significance was confirmed using the Mann-Whitney U test, using the Benjamini-Hochberg method. **(B)** Functional distinctions among TB, NTM, and control patients were assessed based on chosen metabolic pathways and the bacteria linked to these functions through read mapping. **(C)** Heatmap illustrates the average relative abundances of genes involved in Tryptophan metabolism in TB, NTM, and control groups, with statistical significance denoted by Mann-Whitney U test, using the Benjamini-Hochberg method. TDO2 (tryptophan-2,3-dioxygenase 2), KYNU (kynurenine), IDO (indoleamine-2,3-dioxygenase), BNA7 (kynurenine formamidase), and KMO (kynurenine 3-monooxygenase). * FDR-adjusted p < 0.05, ** FDR-adjusted p < 0.01.

Next, we employed HUMAnN3 for functional metagenomic analysis to explore the potential functions of the BALF microbiota in individuals with TB/NTM infections and healthy controls. A comparison between TB/NTM subjects and healthy controls revealed 46 differentially abundant metabolic processes (FDR-adjusted *p* < 0.05, |log2FC| > 1) in the lung microbiota. Among these, 25 metabolic processes were found to be deficient in both TB and NTM groups, including ascorbate and aldarate metabolism, biosynthesis of ansamycins, lysine biosynthesis, fatty acid biosynthesis, glycerophospholipid metabolism, lipoic acid metabolism, lipopolysaccharide biosynthesis, aminoacyl-tRNA biosynthesis, D-glutamine and D-glutamate metabolism, folate biosynthesis, peptidoglycan biosynthesis, ABC transporters, and purine metabolism (**Supplementary Table S5**, **Figure 4A**). In the NTM cohort, we observed a significant elevation in biotin metabolism and tryptophan metabolism, while enrichments in pathways such as D-arginine and D-ornithine metabolism, carbon fixation pathways in prokaryotes, sphingolipid metabolism, and N-glycan biosynthesis were noted but did not reach statistical significance. Furthermore, noteworthy reductions were observed in the NTM group pertaining to propanoate metabolism, glutathione metabolism, and vitamin B6 metabolism. In the TB group, 13 metabolic pathways, including fatty acid metabolism, steroid biosynthesis, taurine and hypotaurine metabolism, valine, leucine, and isoleucine degradation, flavonoid biosynthesis, and glycerolipid metabolism, were enriched, while 12 pathways, such as glycine, serine, and apoptosis, PPAR signaling pathway, and pantothenate and CoA biosynthesis, were significantly downregulated.

When comparing NTM to TB, we found significant enrichment of steroid biosynthesis, taurine and hypotaurine metabolism, steroid hormone biosynthesis, limonene and pinene degradation, flavonoid biosynthesis, and bacterial chemotaxis in the TB group. To determine which bacteria are involved in these pathways, we traced the contributing genes and determined their likely taxonomic origin. The main contributors to taurine and hypotaurine metabolism and steroid hormone biosynthesis were found to be *M. canettii*, MTB, and *M. orygis* (**Figure 4B**). In the NTM group, oxidative phosphorylation and tryptophan metabolism were significantly enriched. Although inositol phosphate metabolism, phosphotransferase system, carbon fixation pathways in prokaryotes, and biotin metabolism showed substantial fold differences between NTM and TB groups, they did not reach statistical significance. To further assess the specific pathways of tryptophan metabolism in NTM, we first investigated the metagenomic gene abundances of three pathways involved in bacterial tryptophan metabolism: the kynurenine pathway, serotonin pathway, and indole pathway. When compared to healthy controls and TB, key genes involved in the kynurenine pathway were upregulated in NTM, including indoleamine-2,3-dioxygenase (IDO) and kynurenine (**Figure 4C**). IDO contributes to the generation of kynurenine-dependent immunosuppression, allowing microbes to persist and evade immune clearance, thus leading to sustained NTM infection (Ouyang et al., 2022).

### Metabolic profiling of BALF in NTM and TB

**Figure 5:**
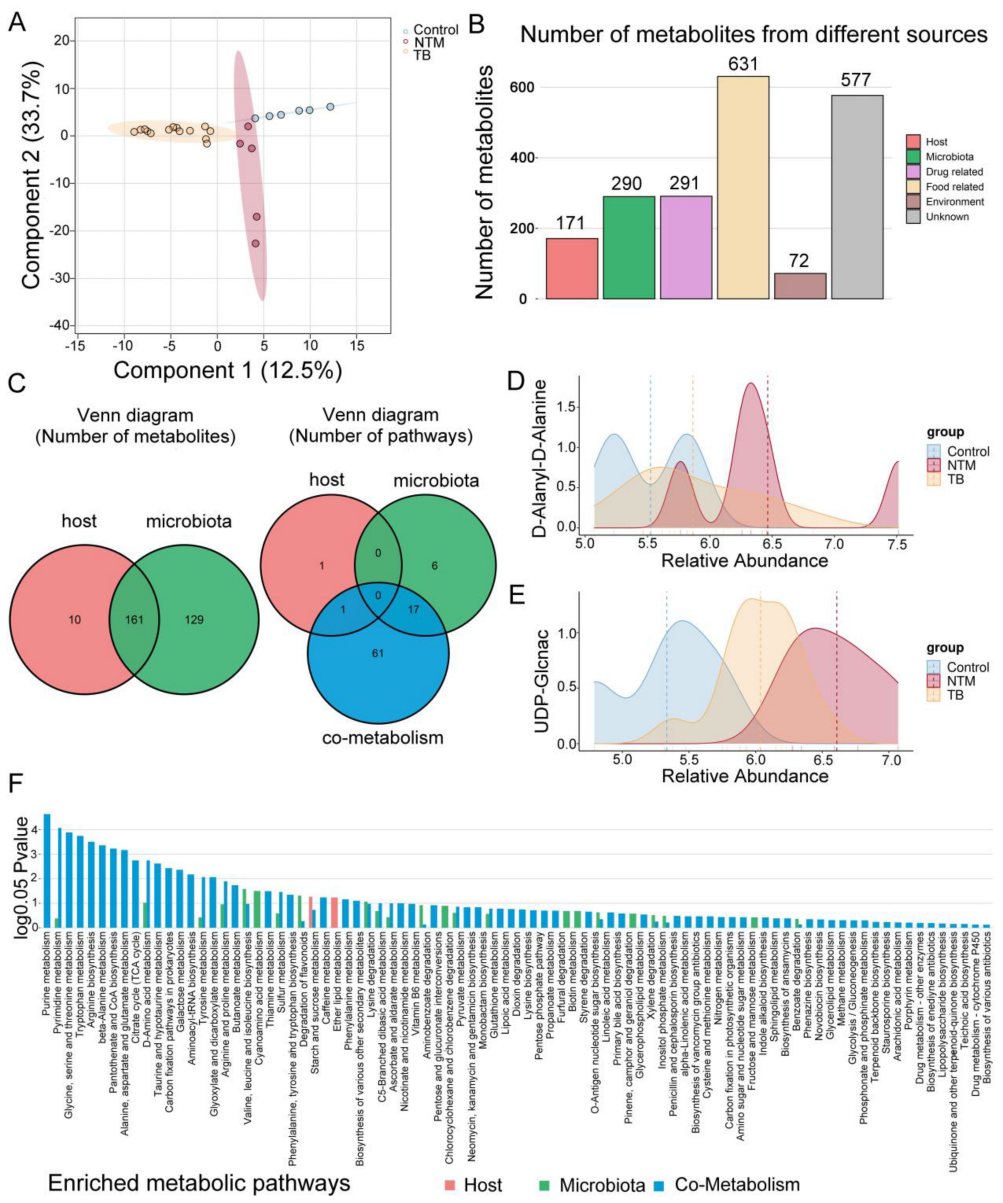
Metabolomic alterations in TB/NTM patients. **(A)** PLS-DA score plots based on the metabolic profiles in BALF samples from control, TB, and NTM group in ES+ and ES−. **(B)** Bar chart illustrates the quantity of metabolites originating from distinct sources. **(C)** Venn diagram illustrates the number of metabolites in the human and bacterial communities, as well as the number of enriched metabolic pathways from origin-based MPEA analysis. **(D, E)** Comparison of **(D)** D-Alanyl-D-Alanine and **(E)** UDP-Glcnac concentrations in the BALF between NTM, TB, and healthy control groups. Significance was confirmed using the Mann-Whitney U test, using the Benjamini-Hochberg method. **(F)** Comparison of differential metabolites obtained from TB and NTM, including subgroups of metabolites from the host, microbiota, or co-metabolism matched to their corresponding KEGG pathways in MPEA. *P* values are calculated using the hypergeometric test.

Considering the dysfunctional BALF microbiota in diseased individuals, our objective was to unravel the intricate interplay between microbiota and host responses in NTM and TB infections. We conducted a comprehensive metabolic analysis on BALF samples from a subset of our cohort: 6 healthy controls, 5 NTM cases (3 intracellular mycobacteria and 2 *M. abscessus*), and 14 TB patients. Using high-throughput Liquid Chromatograph Mass Spectrometer (LC/MS), we identified 1292 metabolites for further investigation (see methods). To distinguish metabolic profiles, we used partial least squares discriminant analysis (PLS-DA) (**Figure 5A**), which clearly differentiated distinct clusters for each group, highlighting the impacts of these infections on metabolic profiles.

For a deeper understanding of metabolite origins and microbial involvement in metabolic processes, we conducted MetOrigin analysis. This analysis categorized the 1292 identified metabolites into four groups: 10 of human origin, 129 of bacterial origin, 161 shared by both bacteria and the host, and the remainder from various external sources (e.g drugs, food, and environment), intersecting with the first three categories (**Figures 5B, C**, **Supplementary Table S6**). Among NTM patients, 442 metabolites exhibited significant differences compared to the control group, while TB patients displayed alterations in 482 metabolites (**Supplementary Table S7**) (FDR-adjusted *p* < 0.05). By comparing NTM and TB, we identified 448 differentially abundant metabolites among the 1292 detected (**Supplementary Table S7**). Remarkably, upregulated D-Alanyl-D-Alanine in NTM group was closely linked to mycobacterial cell wall synthesis (**Figure 5D**). Inhibitors targeting D-Alanyl-D-Alanine are being investigated for TB treatment (Qin et al., 2021), suggesting potential importance for NTM as well. Furthermore, UDP-GlcNAc showed increased levels in both TB and NTM, with a more pronounced increase in NTM compared to TB (**Figure 5E**). UDP-GlcNAc has been identified as a potential target for TB treatment (Patel et al., 2023) and now emerges as a promising candidate for NTM as well. Desthiobiotin, pimelic acid, and 8-amino-7-oxononanoic acid significantly increased in the NTM group, indicating that these microorganisms activate biotin biosynthesis through a series of steps involving pimeloyl-CoA, 8-amino-7-oxononanoate synthase, adenosylmethionine-8-amino-7-oxononanoate aminotransferase, desthiobiotin synthase, and biotin synthase. This aligns with the upregulation of biotin metabolism noted earlier. A recent study by the Rubin lab further supports the critical role of biotin in *M. abscessus* infection, suggesting that targeting the biotin synthesis pathway could effectively inhibit bacterial proliferation (Sullivan et al., 2023). Biotin is an essential coenzyme involved in numerous biochemical reactions across organisms (Daher and Kremer, 2023) and may serve as a potential therapeutic target against NTM infections, warranting further exploration.

To delve deeper into the relevant metabolic pathways, we conducted Metabolite Pathway Enrichment Analysis (MPEA) using differential metabolites and corresponding reference metabolic pathway databases. Based on metabolite tracing, we elucidated the metabolic pathways associated with the host, bacteria, and host-bacteria interactions. (**Figure 5F**, **Supplementary Table S8**). Within bacterial-related metabolic pathways (including those unique to bacteria and those shared with the host), we observed enrichments in pathways such as biotin metabolism, carbon fixation pathways, inositol phosphate metabolism, degradation of flavonoids, tryptophan metabolism, aminoacyl-tRNA biosynthesis, and d-amino acid metabolism. Intriguingly, these unique metabolic pathways closely mirrored functional predictions from metagenomic sequencing. For instance, in the tryptophan metabolism pathway, we noted upregulation of metabolites like tryptophan, kynurenine, anthranilic acid, and formylanthranilic acid in the NTM group compared to healthy controls, while these were downregulated in the TB group. This implies the involvement of NTM-associated microorganisms in the kynurenine pathway. It is worth noting that although indole was upregulated in both NTM and TB groups compared to the control group, it was downregulated in the NTM group when compared to TB, indicating that it might be utilized by NTM bacteria to generate other substances. Furthermore, in the degradation of flavonoids pathway, naringin was upregulated in the TB group, whereas taxifolin, and daidzein were upregulated in the NTM group, indicating their distinct participation in different flavonoid degradation processes. Flavonoids have significant antibacterial effects, including inhibiting energy production in bacterial cells, disrupting cell integrity, and inhibiting enzyme activity within bacteria. Moreover, differences between the two groups emerged in arginine and proline metabolism, with arginine levels decreasing in both the TB and NTM groups compared to controls. Notably, while arginine levels exhibited a more pronounced decrease in the TB group compared to the NTM group, this difference did not reach statistical significance. Arginine plays a pivotal role in nitric oxide (NO) generation (Song et al., 2021), which exerts bactericidal effects against MTB. However, NTM primarily utilizes the ornithine-polyamine/proline pathway for arginine, as opposed to the NO-citrulline cycle. Increasing arginine levels could reshape the lung’s immune defenses against NTM infection.

### Relationships among bacterial species and metabolites

**Figure 6:**
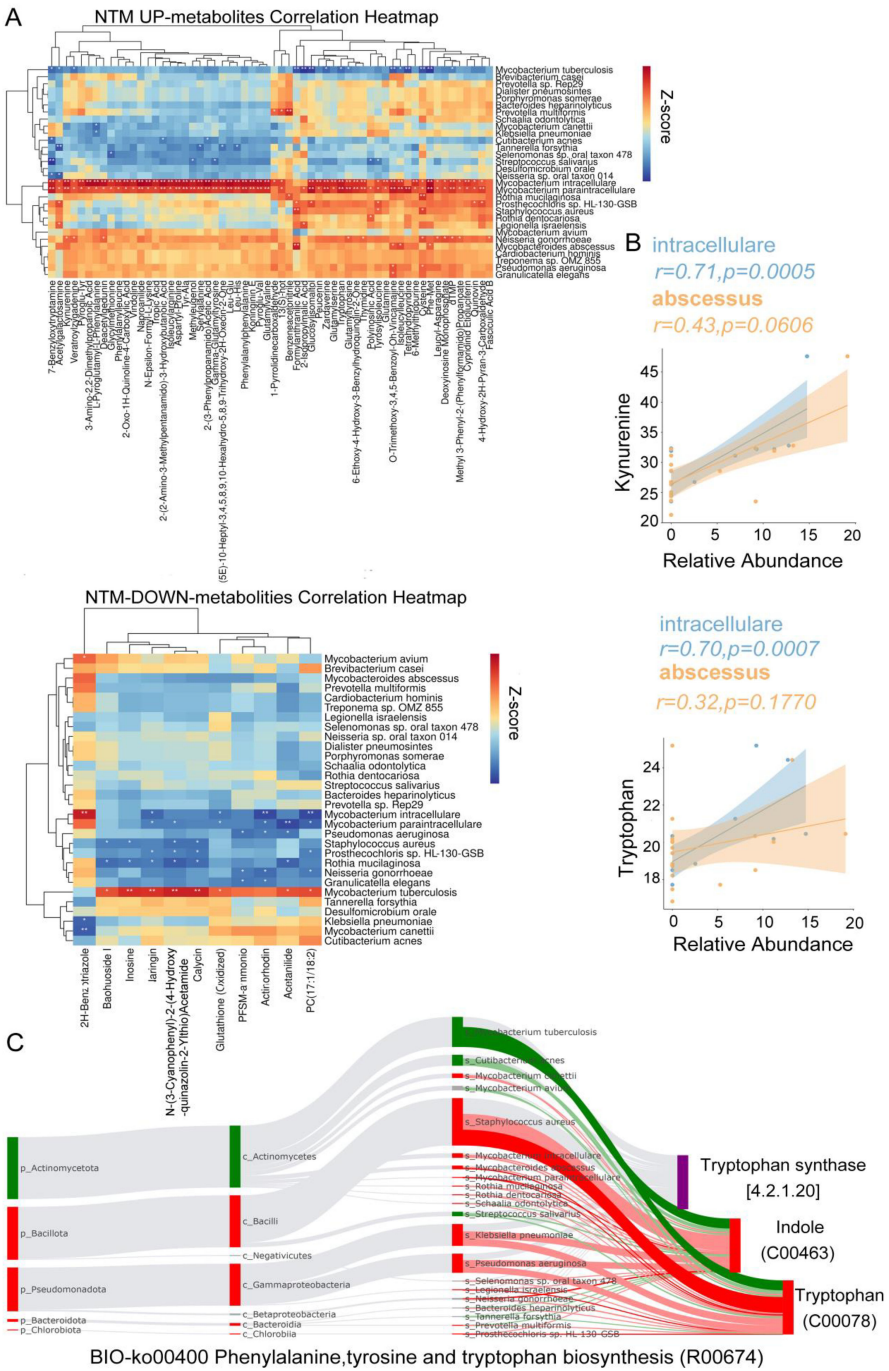
Differential Microbiota Leading to Metabolic Alterations. **(A)** The heatmap illustrates the Spearman correlations between representative differential metabolites of NTM up/down metabolites and 30 distinct differential species (**Figure 2G**) **(B)** The correlation between the concentrations of Tryptophan and Kynurenine and the relative abundances of *M. intracellulare* and *M. abscessus* was assessed. The linear regression trend lines with 95% confidence intervals are depicted around the shaded area. The R and FDR-adjusted p-values were computed to assess the relationship between variables, with FDR-adjusted p-values adjusted for multiple comparisons using the Benjamini-Hochberg method. **(C)** The BIO-Sankey network diagram for the metabolic reaction R00674 in the Phenylalanine, Tyrosine, and Tryptophan biosynthesis pathway. The red/green color of nodes represents upregulation/downregulation, while the red/green bands indicate positive/negative correlations with metabolites.

To elucidate the microbial underpinnings driving the observed variance in metabolite profiles, we identified 226 metabolites exhibiting significant distinctions between the NTM group and both TB and control cohorts. Additionally, a subset of 139 metabolites demonstrated significant disparities between the TB cohort and both NTM and control groups (**Supplementary Table S9**). In the TB group, MTB exhibited a significant positive correlation with naringin, indicating its involvement in flavonoid degradation (**Figure 6A**). MTB also showed a significant positive correlation with glutathione (oxidized), suggesting that as MTB increases, glutathione plays a role in antioxidation and antibacterial activities, converting into glutathione (oxidized). Among the NTM-enriched species, such as *M. paraintracellulare*, and *M. abscessus*, there were significant negative correlations with arginine and inosine. This aligns with previous reports that arginine and inosine can significantly reduce bacterial load in the body and enhance protective T cell responses, producing interferon γ and inducible nitric oxide synthase in NTM-infected mice (Jiang et al., 2022; Kim et al., 2022). Furthermore, we found a significant positive correlation between NTM species (*M. intracellulare* and *M. abscessus*) and the levels of tryptophan and kynurenine (**Figure 6B**). This supports our hypothesis that NTM bacteria may degrade tryptophan, leading to an increase in kynurenine, thereby exerting immunosuppressive effects.

To explore how NTM induce an increase in tryptophan and kynurenine, we continued with Sankey analysis using MetOrgin. We found that *M. intracellulare*, *M. paraintracellulare, M. abscessus*, and *P. aeruginosa* can directly convert indole to tryptophan through tryptophan synthase (**Figure 6C**). This provides an additional supply of this essential amino acid under conditions of tryptophan starvation. The key step is in the conversion of tryptophan to kynurenine by indoleamine-2,3-dioxygenase 1 (IDO1), indoleamine-2,3-dioxygenase 2 (IDO2), and tryptophan-2,3-dioxygenase (TDO) into N-formylkynurenine (Stone and Williams, 2023). IDO1 activity is multifaceted, acting as a double-edged sword (Fiore and Murray, 2021). On one hand, IDO1 depletes tryptophan, starving and reprogramming nutrient-deficient invaders. On the other hand, it generates an immunosuppressive state dependent on kynurenine for microbes that have not been cleared during acute infection or substances capable of reactivating tryptophan biosynthesis.

## Discussion

Despite extensive research on TB infection, our understanding of the molecular intricacies governing NTM infection remains limited. To address this knowledge gap, we utilize integrated metagenomic and metabolomic sequencing to unravel the intricate interplay between pulmonary microbial communities and NTM/MTB infections. Our findings reveal a shared phenomenon in NTM and TB infections characterized by a reduction in α-diversity and a diminished level of microbial interaction within the lung microbiota compared to healthy controls. NTM and TB infections disturb the normal ecological balance and cooperative dynamics within the lung microbiota, facilitating their colonization and infection. Furthermore, our analysis identifies distinct functional gene/pathway and metabolic signatures in the NTM community, particularly in biotin, tryptophan, and arginine metabolism. Notably, enhancement of the tryptophan-kynurenine pathway by Non-Tuberculous Mycobacteria in the lung microenvironment emerges as a significant contributor to immune suppression, potentially conferring a selective advantage to NTM infection in the lungs.

This study represents the first integration of metagenomic sequencing and metabolomic sequencing to comprehensively characterize the lung microbiome in TB and NTM patients. This multifaceted methodology has not only allowed us to identify specific microbial species associated with NTM and TB but also to elucidate the functional implications of these changes at a metabolic level. In the TB cohort, TB exhibits positive correlations with naringin and glutathione (oxidized), suggesting a potential influence over flavonoid degradation and antioxidation, thereby impacting TB’s pathogenesis and the host’s defense machinery. Among NTM-enriched species, the antagonistic correlation of *M. intracellulare*, *M. paraintracellulare*, and *M. abscessus* with arginine and inosine aligns with prior narratives of these metabolites steering immune responses and bacterial eradication during NTM infections. Particularly noteworthy are the affirmative correlations between NTM species and tryptophan and kynurenine, bolstering the contention that NTM bacteria could regulate tryptophan metabolism, consequently increasing kynurenine levels. Kynurenine binds to the aryl hydrocarbon receptor (AHR), resulting in the generation of regulatory IDO1+ dendritic cells (DCs) (Stone and Williams, 2023). This interaction promotes the expansion of regulatory T (Treg) cells and suppresses T helper 17 (TH17) cell responses, ultimately exerting an immunosuppressive effect. These findings intimate that NTM infections orchestrate significant disruptions in the host’s metabolic pathways, with crucial implications on disease progression and immune reactions. Of note, the elevation of D-Alanyl-D-Alanine in both TB and NTM infections opens avenues for potential therapeutic intervention targeting this metabolite. Likewise, the marked surge in UDP-GlcNAc in NTM infections heralds its potential as a therapeutic target for this specific infection. These findings pivot towards novel therapeutic trajectories, aimed at using specific metabolic pathways to augment the host’s defense against TB and NTM infections.

Several studies have highlighted significant differences in lung microbiota between TB/NTM patients and healthy individuals (Gopalaswamy et al., 2020; Schito et al., 2015). However, most of these studies are confined to identifying differential microorganisms through 16s RNA sequencing. This method lacks the capability to distinguish between MTB and NTM, and it doesn’t allow for detailed species or subspecies identification of NTM. Moreover, these studies predominantly focus on TB patients and utilize sputum samples. To date, only one study has conducted metabolic analysis of NTM using sputum samples from patients (Breen et al., 2022). Given that the infection rate of NTM has surpassed that of TB in developed countries (Furuuchi et al., 2019; Winthrop et al., 2020), there is a growing interest in NTM research. In our study, we not only identified specific microbes in TB/NTM patients but also employed SparCC microbial network analysis to evaluate microbiota interactions and identify key microbes. Additionally, we used MetOrigin to reveal the metabolic functions of metabolites from different sources and their correlation with microorganisms. This approach successfully identified crucial bacteria and metabolites in tryptophan metabolism. Importantly, the metagenomic and metabolomic results mutually reinforce each other, ensuring the reliability of the findings. As a result, we identified biotin metabolism, tryptophan metabolism, and arginine metabolism as key factors influencing NTM infection. Furthermore, our study provides a comprehensive view of tryptophan metabolism, detailing the involvement of microorganisms, transformation processes of tryptophan, and its ultimate impact on inhibiting the immune system’s ability to foster NTM infection. Therefore, our study not only identifies key factors involved in NTM infection but also offers valuable methodological references for studying the correlation between diseases and microorganisms.

Also, our study had several limitations. Firstly, the efficacy of epidemic prevention efforts has yielded a significant reduction in the prevalence of TB and NTM infections within China. Consequently, the limited sample size within our study hinders a thorough and all-encompassing understanding of the issue at hand. Secondly, we only use BALF samples, but we believe that it is the best samples for studying the mechanisms underlying the colonization of NTM/TB in lungs except lung tissues. Thirdly, due to the characteristics of contagiousness of NTB and NTM, we are cooperating with the lab at P4 level to confirm the results we received from the study by *in vivo* experiments.

## Conclusion

In summary, our comprehensive multi-omics investigation of lung microbiota and metabolites in NTM and TB infections provides valuable insights into host-microbe interactions and metabolic disruptions. Specifically, NTM induces alterations in lung microbiota and perturbs tryptophan metabolism in NTM patients, leading to increased kynurenine production and the establishment of a specialized niche for sustained NTM infection. The elucidation of these microbial and metabolic dynamics offers a profound understanding of the intricate processes underlying NTM and TB infections, with implications for potential biomarkers and treatment targets.

## Data Availability

The datasets presented in this study can be found in online repositories. The names of the repository/repositories and accession number(s) can be found below: https://ngdc.cncb.ac.cn/gsa/, PRJCA019624.

## References

[B1] Breen P. Zimbric M. Opron K. Caverly L. J. (2022). Sputum metabolites associated with nontuberculous mycobacterial infection in cystic fibrosis. mSphere 7, e0010422. doi: 10.1128/msphere.00104-22 35477313 PMC9241540

[B2] Choi J. Y. Shim B. Park Y. Kang Y. A. (2023). Alterations in lung and gut microbiota reduce diversity in patients with nontuberculous mycobacterial pulmonary disease. Korean J. Intern. Med. 38, 879–892. doi: 10.3904/kjim.2023.097 37867139 PMC10636543

[B3] Cui Z. Zhou Y. Li H. Zhang Y. Zhang S. Tang S.. (2012). Complex sputum microbial composition in patients with pulmonary tuberculosis. BMC Microbiol. 12, 276. doi: 10.1186/1471-2180-12-276 23176186 PMC3541192

[B4] Daher W. Kremer L. (2023). Mycobacterial biotin biosynthesis counters airway alkalinity. Nat. Microbiol. 8, 369–370. doi: 10.1038/s41564-023-01330-0 36797485

[B5] Fiore A. Murray P. J. (2021). Tryptophan and indole metabolism in immune regulation. Curr. Opin Immunol. 70, 7–14. doi: 10.1016/j.coi.2020.12.001 33418116

[B6] Furuuchi K. Morimoto K. Yoshiyama T. Tanaka Y. Fujiwara K. Okumura M.. (2019). Interrelational changes in the epidemiology and clinical features of nontuberculous mycobacterial pulmonary disease and tuberculosis in a referral hospital in Japan. Respir. Med. 152, 74–80. doi: 10.1016/j.rmed.2019.05.001 31128614

[B7] Gopalaswamy R. Shanmugam S. Mondal R. Subbian S. (2020). Of tuberculosis and non-tuberculous mycobacterial infections - a comparative analysis of epidemiology, diagnosis and treatment. J. Biomed. Sci. 27, 74. doi: 10.1186/s12929-020-00667-6 32552732 PMC7297667

[B8] Gupta N. Kumar R. Agrawal B. (2018). New players in immunity to tuberculosis: the host microbiome, lung epithelium, and innate immune cells. Front. Immunol. 10. doi: 10.3389/fimmu.2018.00709 PMC5902499 29692778

[B9] Hu Y. Cheng M. Liu B. Dong J. Sun L. Yang J.. (2020). Metagenomic analysis of the lung microbiome in pulmonary tuberculosis - a pilot study. Emerg. Microbes Infect. 9, 1444–1452. doi: 10.1080/22221751.2020.1783188 32552447 PMC7473061

[B10] Jiang M. Chen Z. G. Li H. Zhang T. T. Yang M. J. Peng X. X.. (2022). Succinate and inosine coordinate innate immune response to bacterial infection. PloS Pathog. 18, e1010796. doi: 10.1371/journal.ppat.1010796 36026499 PMC9455851

[B11] Johansen M. D. Herrmann J. L. Kremer L. (2020). Non-tuberculous mycobacteria and the rise of Mycobacterium abscessus. Nat. Rev. Microbiol. 18, 392–407. doi: 10.1038/s41579-020-0331-1 32086501

[B12] Kang S. Y. Kim H. Jung S. Lee M. Lee S. P. (2021). The lung microbiota in Korean patients with non-tuberculous mycobacterial pulmonary disease. BMC Microbiol. 21, 84. doi: 10.1186/s12866-021-02141-1 33736609 PMC7977250

[B13] Kim Y. J. Lee J. Y. Lee J. J. Jeon S. M. Silwal P. Kim I. S.. (2022). Arginine-mediated gut microbiome remodeling promotes host pulmonary immune defense against nontuberculous mycobacterial infection. Gut Microbes 14, 2073132. doi: 10.1080/19490976.2022.2073132 35579969 PMC9116420

[B14] Krishna P. Jain A. Bisen P. S. (2016). Microbiome diversity in the sputum of patients with pulmonary tuberculosis. Eur. J. Clin. Microbiol. Infect. Dis. 35, 1205–1210. doi: 10.1007/s10096-016-2654-4 27142586

[B15] Lachmandas E. Heuvel den van C. N. Damen M. S. Cleophas M. C. Netea M. G. Crevel van R. (2016). Diabetes mellitus and increased tuberculosis susceptibility: the role of short-chain fatty acids. J. Diabetes Res. 2016, 6014631. doi: 10.1155/2016/6014631 27057552 PMC4709651

[B16] Lawani M. B. Morris A. (2016). The respiratory microbiome of HIV-infected individuals. Expert Rev. Anti-Infect. Ther. 14, 719–729. doi: 10.1080/14787210 27348261 PMC4980914

[B17] Naito K. Noguchi S. Yatera K. Kawanami T. Yamasaki K. Fukuda K.. (2020). Coinfection with multiple nontuberculous mycobacteria as a possible exacerbating factor in pulmonary nontuberculous mycobacteriosis: clone library analysis using the 16S ribosomal RNA gene. Chest 158, 2304–2313. doi: 10.1016/j.chest.2020.06.027 32599068

[B18] Ouyang L. Yu C. Xie Z. Su X. Xu Z. Song P.. (2022). Indoleamine 2,3-dioxygenase 1 deletion-mediated kynurenine insufficiency in vascular smooth muscle cells exacerbates arterial calcification. Circulation 145, 1784–1798. doi: 10.1161/CIRCULATIONAHA.121.057868 35582948 PMC9197997

[B19] Patel Y. Soni V. Rhee K. Y. Helmann J. D. (2023). Mutations in rpoB That Confer Rifampicin Resistance Can Alter Levels of Peptidoglycan Precursors and Affect beta-Lactam Susceptibility. mBio 14, e0316822. doi: 10.1128/mbio.03168-22 36779708 PMC10128067

[B20] Qin Y. Xu L. Teng Y. Wang Y. Ma P. (2021). Discovery of novel antibacterial agents: Recent developments in D-alanyl-D-alanine ligase inhibitors. Chem. Biol. Drug Des. 98, 305–322. doi: 10.1111/cbdd.13899 34047462

[B21] Schito M. Migliori G. B. Fletcher H. A. McNerney R. Centis R. D'Ambrosio L.. (2015). Perspectives on advances in tuberculosis diagnostics, drugs, and vaccines. Clin. Infect. Dis. 61, S102–S118. doi: 10.1093/cid/civ609 PMC4583570 26409271

[B22] Segal L. N. Clemente J. C. Li Y. Ruan C. Cao J. Danckers M.. (2017). Anaerobic bacterial fermentation products increase tuberculosis risk in antiretroviral-drug-treated HIV patients. Cell Host Microbe 21, 530–537.e4. doi: 10.1016/j.chom.2017.03.003 28366509 PMC5465639

[B23] Song H. Hewitt O. H. Degnan S. M. (2021). Arginine biosynthesis by a bacterial symbiont enables nitric oxide production and facilitates larval settlement in the marine-sponge host. Curr. Biol. 31, 433–437.e3. doi: 10.1016/j.cub.2020.10.051 33220182

[B24] Stone T. W. Williams R. O. (2023). Modulation of T cells by tryptophan metabolites in the kynurenine pathway. Trends Pharmacol. Sci. 44, 442–456. doi: 10.1016/j.tips.2023.04.006 37248103

[B25] Sulaiman I. Wu B. G. Li Y. Scott A. S. Malecha P. Scaglione B.. (2018). Evaluation of the airway microbiome in nontuberculous mycobacteria disease. Eur. Respir. J. 52, 1800810. doi: 10.1183/13993003.00810-2018 30093571

[B26] Sullivan M. R. McGowen K. Liu Q. Akusobi C. Young D. C. Mayfield J. A.. (2023). Biotin-dependent cell envelope remodelling is required for Mycobacterium abscessus survival in lung infection. Nat. Microbiol. 8, 481–497. doi: 10.1038/s41564-022-01307-5 36658396 PMC9992005

[B27] Varley C. D. Winthrop K. L. (2022). Nontuberculous mycobacteria: diagnosis and therapy. Clin. Chest Med. 43, 89–98. doi: 10.1016/j.ccm.2021.11.007 35236564

[B28] Wang D. M. Liu H. Zheng Y. L. Xu Y. H. Liao Y. (2023). Epidemiology of nontuberculous mycobacteria in tuberculosis suspects, southwest of China 2017-2022. Front. Cell. Infect. Microbiol. 13, 1282902. doi: 10.3389/fcimb.2023.1282902 PMC10644767 38029240

[B29] Wang S. Xing L. (2023). Metagenomic next-generation sequencing assistance in identifying non-tuberculous mycobacterial infections. Front. Cell. Infect. Microbiol. 13. doi: 10.3389/fcimb.2023.1253020 PMC10500063 37719673

[B30] Winthrop K. L. Marras T. K. Adjemian J. Zhang H. Wang P. Zhang Q. (2020). Incidence and prevalence of nontuberculous mycobacterial lung disease in a large U.S. Managed care health plan 2008-2015. Ann. Am. Thorac. Soc 17, 178–185. doi: 10.1513/AnnalsATS.201804-236OC 31830805 PMC6993793

[B31] Wu J. Liu W. He L. Huang F. Chen J. Cui P.. (2013). Sputum microbiota associated with new, recurrent and treatment failure tuberculosis. PloS One 8, e83445. doi: 10.1371/journal.pone.0083445 24349510 PMC3862690

[B32] Yamasaki K. Mukae H. Kawanami T. Fukuda K. Noguchi S. Akata K.. (2015). Possible role of anaerobes in the pathogenesis of nontuberculous mycobacterial infection. Respirology 20, 758–765. doi: 10.1111/resp.12536 25824634

[B33] Zhou Y. Lin F. Cui Z. Zhang X. Hu C. Shen T.. (2015). Correlation between either cupriavidus or porphyromonas and primary pulmonary tuberculosis found by analysing the microbiota in patients' Bronchoalveolar lavage fluid. PloS One 10, e0124194. doi: 10.1371/journal.pone.0124194 26000957 PMC4441454

